# Intra-tumoral distribution of Ki-67 and Cyclin D1 in ER+ mammary carcinoma: quantitative evaluation

**DOI:** 10.4314/ahs.v21i1.7

**Published:** 2021-03

**Authors:** Mohammedi Latifa, Djillali Doula Fatima, Mesli Farida, Senhadji Rachid

**Affiliations:** Nature and Life Sciences Faculty, University of Oran 1 Ahmed Ben Bella, BP 1524 El M'naouer, Oran 31000, Algeria

**Keywords:** Breast cancer, Cyclin D1, ER+, Intra-tumoral heterogeneity, Ki-67

## Abstract

**Background:**

In spite of the strong evidence demonstrating the role of overexpression of Ki-67 and Cyclin D1 markers in breast carcinomas, clinical and pathological data remain to be discussed. This can be explained partly by intratumor heterogeneity.

**Objectives:**

To define the prevalence and clinical significance of Ki-67 and Cyclin D1 overexpression in primary breast tumors ER positive, while highlighting the existence of intratumor heterogeneity in this type of cancer

**Materials and methods:**

51 ER positive breast cancer tumors were used to evaluate the intratumoral distribution of Ki-67 and Cyclin D1 expression. Image acquisition and visualization of the markers were performed by optical microscopy and stereology sampling method.

**Results:**

The mean Ki-67 labeling index was distributed heterogeneously in the same tumor, from 20.67±6.87 to 45.10±10.65. The coefficient of variation (COV) revealed dispersion values between 13.4% and 42.9%. Associated with positive ER status, all the tumors presented a Cyclin D1 expression with a COV varying between 19% and 28.5% and a mean labeling index fluctuating between 19.40±4.42 and 41.64±10.08 within the same patient showing important intratumor heterogeneous distribution.

**Conclusion:**

In this study, we have adopted a strictly quantitative approach to evaluate and demonstrate intratumor heterogeneity. This establishes one of the main factors for poor response to cancer therapy. To achieve this, intratumor heterogeneity should be usually definable and quantifiable but this domain awaits future progress and methods need to move towards a better understanding of molecular and cellular mechanisms that initiate and maintain this tumor heterogeneity.

## Introduction

The breast cancer is a histologically and clinically heterogeneous disease. The intra-tumoral heterogeneity is due to phenotypically diverse cancer stem cells which can be a crucial matter in terms of therapeutic responses^1^. In addition to the known histo-morphological criteria, the detection and the quantification of this intratumor heterogeneity will enable to determine groups of patients with a more accurate prognosis^2^. Spatial distribution of Ki-67 is investigated, given the importance of Ki-67 as a prognostic parameter and its contribution in treatment decisions^3^. The oncogenic properties of Cyclin D1 in breast cancer in particular ER+ invasive ductal carcinoma (IDC) have been established in various studies^4^–^6^. Cyclin D1 overexpression has been reported in 40% to 90% of invasive breast cancer^7^–^9^. The aim of this preliminary study was to define the prevalence and clinical significance of Ki-67 and Cyclin D1 overexpression in primary ER positive invasive breast cancer, while highlighting the existence of intratumor heterogeneity in this type of cancer.

## Material and methods

Fifty one (51) tumor specimens from female patients with grade III invasive ductal carcinoma (IDC) were used for this study. Paraffin-embedded tumor samples and medical data of the selected patients were obtained from the Regional Military University Hospital of Oran. Labeling index (LI) (i.e. percentage of positive stained cells) and coefficient of variation (SD/mean, COV) of Ki-67 and Cyclin D1 expression were determined to measure the dispersion. Scoring of the Ki-67 and Cyclin D1 reactivity was performed by using the Allred method, which classifies tumors into three groups: negative/weak (scores 0–2), moderate (scores 3–5) and strong (scores 6–8)^10^.

Paraffin sections were mounted on APES (2% 3′-aminopropyltriethoxysilane) coated slides. A monoclonal Mouse antibody Anti-Human Cyclin D1 clone DCS-6, DAKO (provided in liquid form as tissue culture supernatant in 0.05 mol/L Tris-HCl, pH 7.6 and 0.015 mol/L sodium azide) and Mib-1, mouse monoclonal antibody ready-to-use (DAKO) were used. The indirect avidin-biotin immunoperoxidase technique was used to demonstrate antibody binding sites. Finally, the sections were lightly counterstained in hematoxylin.

Tumor samples used to assess intratumor heterogeneity (ITH) were selected by random sampling method^11^. Full cell count method was done by sweeping the slide from the right to the left then from the top to the down^12^. Slides were subdivided in fields (images) delimited by the microscope grads. To avoid oversampling, the number of cells was estimated by two-dimensional counting rule described by Gundersen (1977)^13^. In addition to cells within the frame, all cells intersected by the upper and right border are counted and all those intersected by the lower and left border are disregard, and any cell hit by the upper left corner are counted and those hit by the lower right corner are disregard. Slides were reviewed using an Optical microscope (Olympus, CH20 BIMF200) at 40X objective, equipped with a camera (OPTIKA Vision Lite 1.04 OPTIKAM B5) connected to a computer.

## Results

**Figure 1:**
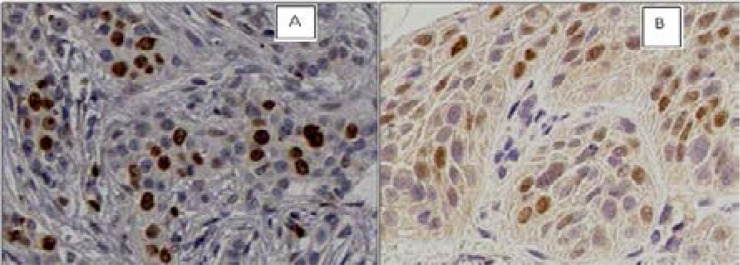
Representative Nuclear staining results from Mayer's hematoxylin coloration and immunohistochemistry for Ki-67 (A) and Cyclin D1(B), x 400.

Ki-67 and Cyclin D1 slides (Figure 1), showed positive nuclear staining but uniformly from one field to another. Positive perinuclear and sometimes nucleolar reactions were seen with perinuclear reinforcement. The number of microscopic fields analyzed per slide were estimated between 8 and 13. In total, 537 fields have been treated and 52700 cells have been counted (Table 1).

**Table 1:** Data of image acquisition showing the field's number and counted cells

Number of Slides	Number of Fields/Slide	Total number of fields	Number of cells
9	12	108	12860
9	11	99	9240
8	9	72	6220
8	8	64	6580
9	10	90	7470
8	13	104	10330

Total		537	52700

**Figure 2:**
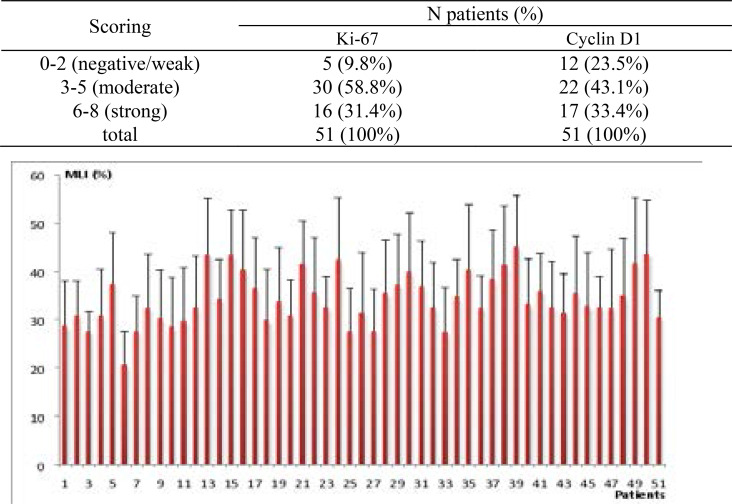
Mean labeling indexes (MLI %) for Ki-67 and standard deviation by patient.

**Figure 3:**
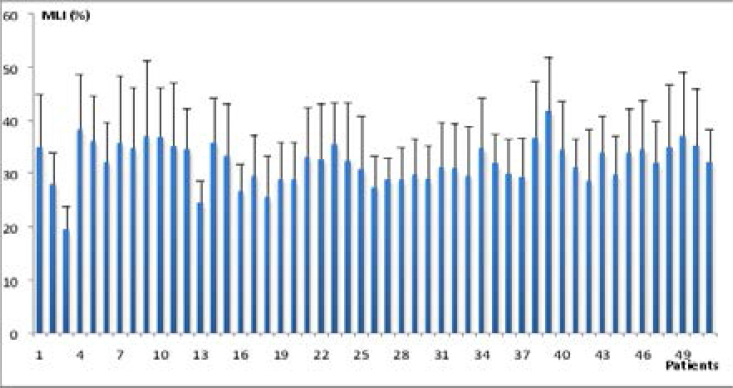
Mean labeling indexes (MLI %) for Cyclin D1 and standard deviation by patient.

Ki-67 and Cyclin D1 showed variations in expression levels in the same tumor. The maximal (Mx) and minimal (Mn) of mean labeling indexes (MLI) for Ki-67 were found in patients 39 and 6 with respective values of 45.10±10.65% and 20.67±6.87% (Figures 2), the median value was 32.65%. As for Cyclin D1, the maximal mean labeling index was detected in the patient 39 with a value of 41.64±10.08%, the minimal value was found in patient 3 (19.40±4.42%) (Figure 3), the median value was 32.12%.

Ki-67 and Cyclin Dl expressions as detected by immunohistochemistry were seen in 46 (90.2%) and 39 (76.5%) cases respectively. Cases were categorized in two groups, moderate 30 (58.8%) and 22 (43.1%) and strong 16 (31.4%) and 17 (33.4%) (Table 2).

**Table 2:** Ki-67 and Cyclin D1 scoring expression

Scoring	N patients (%)	

	Ki-67	Cyclin D1
0–2 (negative/weak)	5 (9.8%)	12 (23.5%)
3–5 (moderate)	30 (58.8%)	22 (43.1%)
6–8 (strong)	16 (31.4%)	17 (33.4%)
total	51 (100%)	51 (100%)

Thirty four (67%) slides in which both Ki-67 and Cyclin D1 were positives were used to evaluate the markers expression by immunohistochemistry. Overall, 17 (33%) cases were negatives with 5 (9.8%) cases negative for Ki-67 and 12 (23.5%) cases negative for Cyclin D1. Positives cases were categorized into two groups; moderate 30 (58.8%) and 22 (43.1%) for Ki-67 and Cyclin D1 respectively and strong 16 (31.4%) and 17 (33.4%) for Ki-67 and Cyclin D1 (Table 2).

**Figure 4:**
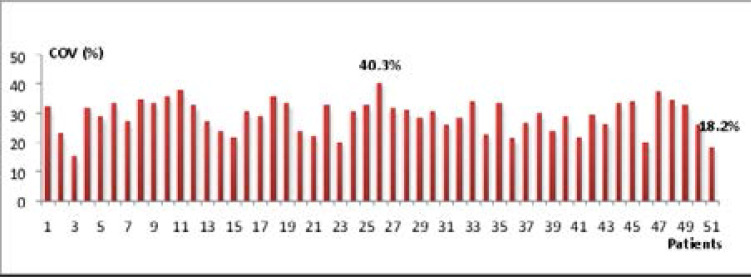
Coefficient of variation (COV%) of Ki-67 labeling index.

**Figure 5:**
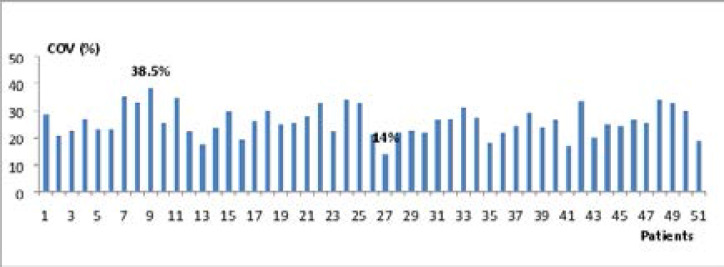
Coefficient of variation (COV%) of Cyclin D1labeling index.

The estimation of COV showed a dispersion of labeling within the same patient (Figures 4 and 5). The COV of Ki-67 labeling index ranged from 18.2% in patient 51 to 40.3% in patient 26 with Cyclin D1 labeling index also showed a wide dispersion within the same tumor with COVs from one patient to another ranging from 14% to 38.5%.

## Discussion

The present study illustrates the existence of a heterogeneous distribution of Ki-67 and Cyclin D1 expression within and among invasive ductal carcinomas (IDC) patients. The Ki-67 cut-off established by the St Gallen International Expert Consensus during which the majority of panelists voted that a threshold of ≥ 20 % was indicative of high Ki-67 status^14^. In our series, the median Ki-67 and Cyclin D1 values were 32.65% and 32.12% respectively, which means that the 51 tumors studied had a great proliferative activity reflecting the tumor aggressiveness of IDC.

Our study shows that 90.2% of tumors had a moderate/strong Ki-67 expression, this is in accordance with a previous study^15^ which reported that 80.7% of cases presented a high expression of Ki-67. As to Cyclin D1, 76.5% show a moderate/strong expression. This result is in accordance with this observed in previous studies^16^–^17^ showing high proliferative activity of Cyclin D1 in ER+ IDC and advocating including Cyclin D1 as an independent prognostic factor to predict the risk of mortality in ER+ IDC patients. This result can be also explained by the positive regulation of ER by Cyclin D1 in breast cancer cell lines where Cyclin D1 has been shown to join directly and activate the estrogen receptor alpha (ER)^18^. This finding has the potential to add a crucial dimension to our understanding of mammary carcinogenesis. Direct targeting of Cyclin D1 should be included in therapeutic protocols. because It's a promising pathway in view of the development of selective treatment for patients and should increase the chances of therapeutic success in these tumors.

Based on the evaluation of the COV to characterize the intratumor heterogeneity of Ki-67 expression in IDC, our data are consistent with the results of Senhadji (2004)^19^ who found fluctuating COV values between 1.94% and 35.9%. Similarly, for Cyclin D1, the results found confirming intratumor heterogeneity, are in line with those found in earlier study^17^ which reported that the staining intensity varied within the individual tumor and from cell to cell within the same tumor. In the present study, the dispersion estimated by the COV is very high in some cases, 40.3% for the Ki-67 and 38.5% for Cyclin D1. This shows the great variability in the distribution of labeling reflecting the heterogeneity of tumors studied. The intratumor heterogeneity shown in this study has been also confirmed by other researchers^20^,^21^,^22^. These findings recommend exploring the whole tumor and not only a part of it, because intratumor heterogeneity can lead to underestimation of the tumor genomics landscape portrayed from single tumor-biopsy samples and may present major challenges to personalized-medicine and biomarker development.

## Conclusion

Our work is essentially a quantitative approach demonstrating tumour heterogeneity by the markers Ki-67 and Cyclin D1 expression. We confirm that the two biomarkers are expressed and distributed differently between microscopic fields within the same patient. This finding demonstrates the presence of intratumor heterogeneity indicating that ER+ IDC is often a mixture of multiple genotypically distinct cell populations. This explains one of the main reasons for poor response to oncological therapy^22^ and thus it is important to integrate intratumor heterogeneity into cancer care. However, this domain awaits future studies and application of novel techniques like genomics and bioinformatics for better understanding of the basic molecular and cellular mechanisms that initiate and maintain this tumor heterogeneity.
